# Paeonol Improves Cardiac Remodelling in MI Mice by Suppressing NOX2 mRNA Expression to Mitigate Oxidative Stress and Mitochondrial Dysfunction

**DOI:** 10.1111/jcmm.70563

**Published:** 2025-05-08

**Authors:** Yun Liu, Zhiming Wu, Xiaoping jin, Meili Ji, Tianyi Huang, Peina Meng, Tian Xu, Wei You, Yanfang Zhao, Fei Ye, Xiangqi Wu

**Affiliations:** ^1^ Department of Cardiology Nanjing First Hospital, Nanjing Medical University Nanjing China; ^2^ Department of Cardiology The Eighty‐First Hospital of PLA Affiliated With Anhui Medical University Nanjing China; ^3^ Department of Geriatric Nanjing First Hospital, Nanjing Medical University Nanjing China

**Keywords:** mitochondrial dysfunction, NOX2, oxidative stress, paeonol

## Abstract

Myocardial infarction (MI), a primary contributor to mortality from cardiovascular diseases, continues to pose a significant challenge in clinical treatment. In this study, our objective was to investigate the cardioprotective effects of paeonol (PAE) on mice with MI, and to delve into the precise mechanisms underlying these effects. We developed the MI model by ligating the left anterior descending artery in mice and replicated this model in vitro by stimulating H9C2 cells with levarterenol (LN). Cardiac function, infarct size, cardiomyocyte size, apoptosis, and mitochondrial structure were evaluated through echocardiography, Masson's trichrome staining, WGA staining, TUNEL assay, and electron microscopy, respectively. Colorimetry, Western blotting, flow cytometry, RT‐PCR, and the dual‐luciferase reporter assay were employed to explore the underlying mechanisms. Compared with the model group, PAE significantly ameliorated cardiac dysfunction and hypertrophy, diminished infarct size, cardiomyocyte hypertrophy, and apoptosis, mitigated mitochondrial structural damage, lowered levels of malondialdehyde and NOX2, reduced ROS production, and NOX activity, while enhancing the activities of T‐SOD, GSH‐PX, and mitochondrial complexes I‐V in mice with MI or H9C2 cells subjected to LN intervention. Ultimately, PAE was found to negatively regulate the transcription of NOX2 mRNA in H9C2 cells, partly through inhibition of phospho‐STAT3‐Y705 protein expression. These results imply that PAE's transcriptional inhibition of NOX2 mRNA expression primarily confers a cardioprotective effect, mitigating myocardial remodelling following MI by improving oxidative stress and mitochondrial dysfunction. This indicates that PAE holds therapeutic promise for the treatment of patients post‐MI.

## 1 Introduction

Myocardial infarction (MI), a primary cause of death in cardiovascular diseases, continues to pose a significant challenge in clinical treatment [1]. MI is the result of myocardial hypoxia and cardiomyocyte energy failure. Prolonged myocardial ischemia, often due to coronary artery occlusion, can exacerbate the condition of MI [2]. Cardiac remodelling ensues post‐MI, ultimately resulting in chronic heart failure—a complex issue [3]. Late cardiac remodelling following MI predominantly manifests as cardiomyocyte hypertrophy, apoptosis and diffuse interstitial fibrosis, typically occurring within a few weeks [4]. To date, the classic therapeutic drugs include the following: Angiotensin‐Converting Enzyme Inhibitors (ACEIs), Angiotensin II Receptor Antagonists (ARBs), and Angiotensin Receptor Neprilysin Inhibitors (ARNIs); β‐blockers; Aldosterone Receptor Antagonists; and Sodium‐Glucose Cotransporter‐2 Inhibitors [5]. These medications have the potential to lower the likelihood of hospital readmission and cardiovascular mortality among individuals suffering from heart failure subsequent to a MI. Nonetheless, certain patients continue to face a grim prognosis, underscoring the necessity for the development of innovative drugs that target novel therapeutic pathways [6].

PAE is a principal active compound extracted from the extensively utilised Chinese herbal remedy, *Paeonia suffruticosa* [7]. It is widely recognised that PAE possesses a range of pharmacological properties, encompassing anti‐inflammatory, anti‐oxidative, and anti‐apoptotic effects, among others [7, 8]. PAE has been demonstrated to exert a cardioprotective effect across various disease models, both in vivo and in vitro, including the transverse aortic constriction model, the doxorubicin‐induced cardiotoxicity model, and the diabetic cardiomyopathy model, among others [9, 10]. Li et al. reported that the combination of PAE and Danshensu exhibits a cardioprotective effect against isoproterenol‐induced MI in rats. This effect is mediated through the activation of the nuclear factor‐erythroid 2‐related factor 2/heme oxygenase‐1 signalling pathway and partially involves the phosphatidylinositol 3 kinase/protein kinase B cell survival signalling pathway [11]. Tsai and his colleagues have proposed that the cardioprotective effect of PAE during ischemia/reperfusion injury could stem from its modulation of the crosstalk between apoptotic and autophagic signalling pathways, thereby suppressing apoptosis and autophagic cell death [12]. Hu and her team have indicated that PAE and paeoniflorin may play a protective role against acute myocardial infarction [complete ligation of the left anterior descending artery (LAD) for 5 days] by participating in the immune process, inhibiting inflammatory responses, and regulating energy metabolism [13]. In summary, these studies have demonstrated that PAE exerts a protective effect against myocardial ischemic injury induced by isoproterenol (ISO) and acute myocardial ischemic conditions. To date, no data have been reported on the cardioprotective effects of PAE on chronic cardiac remodelling following myocardial infarction, nor on its precise mechanisms.

Consequently, in this study, we conducted a complete ligation of the LAD in mice for a duration of 4 weeks to establish a model of chronic myocardial ischaemic injury. We then observed the protective effects of PAE within this model and proceeded to delve into its precise mechanism of action.

## 2 Materials and Methods

### 2.1 Mice

Mice possessing a C57BL/6 genetic background were group‐housed under an 8–16 h daily light–dark cycle, with unrestricted access to food and water, in compliance with the mouse welfare and ethical guidelines of Nanjing Hospital, affiliated with Nanjing Medical University (Nanjing, China). The mice, with body weights ranging from 20 to 30 g, were procured from the Qinglongshan Animal Breeding Farm located in Nanjing, China. The experimental protocol received approval from the Institutional Animal Care and Use Committee of Nanjing Medical University (approval no. IACUC‐2004016) and was conducted in strict accordance with the National Institutes of Health's Guide for the Care and Use of Laboratory Animals.

### 2.2 Establishment of the MI Model

The MI was generated using a method that slightly modified a previously reported procedure in mice [14]. In brief, the mice were administered intraperitoneal anaesthesia using pentobarbital at a dosage of 100 mg/kg. Subsequently, a 20‐gauge polyethylene catheter was inserted into the trachea, and a small animal ventilator (ALC‐V8S, manufactured by Shanghai Alcott Biotech Co. Ltd.) was employed to deliver positive pressure ventilation at a volume of 2–3 mL per cycle, with a respiratory rate set at 130 cycles per minute. Upon accessing the thoracic cavity at the fourth rib level and along the left sternal border, the LAD coronary artery was ligated using a 7–0 silk suture, positioned 2–3 mm from the apex of the left auricle. The chest wall was sutured closed using a continuous 6–0 prolene thread, followed by the application of a 4–0 polyester thread to close the skin. The sham operation procedure was identical to the previously described MI‐induced protocol, with the exception of the LAD artery ligation. Upon completion of the surgery, carprofen (1 mg/kg) was administered subcutaneously to alleviate pain in the mice. Furthermore, administering enrofloxacin via intraperitoneal injection at a dosage of 10 mg/kg to mice serves to prevent incisional infections.

### 2.3 PAE Treatment

Approximately 2‐month‐old male mice were randomly and evenly assigned to one of the following three groups: (1) Sham‐operation group (*N* = 15); (2) MI group (*N* = 15); (3) MI + PAE group (*N* = 15). Post‐operation, PAE was administered intraperitoneally daily for a period of 4 weeks. The recommended injectable dose of PAE, based on preliminary testing, is 100 mg/kg/d. PAE (purity ≥ 98%, molecular formula C9H10O3, molecular weight 166.18, supplied by Nanjing DASF Bio‐Technology Co. Ltd., Nanjing, China) was dissolved in sterile saline. The molecular structure of PAE is depicted in Figure 5. Both the sham‐operation and MI groups received an intraperitoneal administration of the same dose of solvent as the MI + PAE group. Moreover, to test the effect of PAE (100 mg/kg/day) on normal mice over a period of 4 weeks, 12 2‐month‐old mice were randomly assigned to two groups: a control group (*N* = 6) and a control + PAE group (*N* = 6).

Upon completion of heart conservation, measurements of heart weight (HW) and tibial length (TL) were taken, and the ratios of HW to body weight (BW) and HW to TL were subsequently calculated. The supernatant was isolated from the heart tissue through centrifugation at 3000 rpm for a duration of 15 min.

### 2.4 Echocardiography [14]

An echocardiographic examination was conducted on the 28th day post‐MI induction, utilising a Vevo 2100 UBM system (Visual Sonics, Toronto, Canada) equipped with a 30‐MHz transducer for noninvasive transthoracic echocardiography. Two‐dimensional guided M‐mode recordings were captured. Eventually, the left ventricular internal diameter at end‐diastole (LVID; d), the left ventricular internal diameter at end‐systole (LVID; s), the left ventricular fractional shortening (LVFS), and the left ventricular ejection fraction (LVEF) were calculated.

### 2.5 Cell Culture

The H9C2 cell line, originating from rat embryonic ventricular cardiomyocytes, was procured from Sigma‐Aldrich (Merck KGaA, Darmstadt, Germany). It has been maintained in DMEM supplemented with 10% (v/v) fetal bovine serum (FBS), 100 units/mL penicillin, and 0.1 mg/mL streptomycin, under an atmosphere of 5% CO_2_ at a temperature of 37°C. Subsequently, the cells were allocated into three distinct groups: the control group, which was treated with dimethyl sulfoxide (DMSO) alone (*N* = 11), the LN group, which received a 2 μM concentration (*N* = 11), and the LN (2 μM) plus PAE (100 μM) group, also with *N* = 11. Both LN and PAE were dissolved in DMSO. The three groups were subjected to the aforementioned method of intervention for a duration of 48 h. However, the group receiving both LN and PAE was subjected to PAE treatment for a duration of 24 h. Furthermore, to assess the effect of PAE (100 μM) on H9C2 cells over a 24‐h period, we randomly assigned the cells into two groups: the control group (*N* = 5) and the control + PAE group (*N* = 5). LN was acquired from Alfa Aesar Chemical Co. Ltd.

Finally, the cells were harvested for subsequent biochemical assays, western blot analysis, and immunofluorescence staining. Specifically, the phalloidin immunofluorescence detection kit was sourced from Solarbio Technology in Beijing, China, while the TUNEL immunofluorescence detection kit was obtained from Roche Molecular Biochemicals in Mannheim, Germany.

### 2.6 Histology

The protocols for Masson's trichrome staining and immunofluorescence (IF) were executed in accordance with previously reported methods [15]. In brief, the heart samples were initially rinsed with ice‐cold phosphate‐buffered saline (PBS) and subsequently fixed in 4% paraformaldehyde at 4°C. The samples underwent sequential processing steps: (a) a 30‐min wash in PBS at 4°C; (b) 15‐min immersions in 30%, 50%, 75%, and 85% ethanol solutions, followed by two 10‐min incubations in 95% and 100% ethanol at room temperature (RT); (c) three 10‐min incubations in xylene at RT; (d) a 20‐min incubation in a 1:1 paraffin/xylene mixture at 65°C; and (e) three 30‐min incubations in fresh paraffin at 65°C. The heart tissue samples underwent paraffin embedding and were subsequently sliced to a thickness ranging from 4 to 6 μm. Following this, the sections were prepared for Masson staining.

The IF staining procedure was conducted using wheat germ agglutinin (WGA) from BIOTIUM and 4',6‐diamidino‐2‐phenylindole (DAPI) from CST, both at room temperature for a duration of 4 h. Subsequently, five fields from the sample were examined under a confocal microscope (Carl Zeiss, Germany).

Apoptosis was assessed by Terminal‐deoxynucleotidyl Transferase Mediated Nick End Labeling (TUNEL) assay using an in situ cell death detection kit (Roche Applied Science) [16]. The fibrotic region, cardiomyocyte area, and apoptotic cells were quantified using a 400× magnification lens and averaged across 5 high‐power fields after calculation.

A minute (approximately 1 mm^3^) sample of cardiac tissue was extracted from the border zone of the infarct region and promptly fixed with 2.5% glutaraldehyde for 2–4 h, subsequently fixed with 2% OsO_4_ for 1 h, and embedded in Epon 812. Then, the slides were double‐stained with uranium acetate and lead citrate and observed and photographed with an HT7700 SS/FEI Tecnai G20 (Hitachi Limited).

### 2.7 Spectrophotometry Determination [17]

The analysis kits for total‐superoxide dismutase (T‐SOD), glutathione peroxidase (GSH‐PX), malonaldehyde (MDA), mitochondrial complexes (I, II, III, IV, and V), adenosine triphosphate (ATP), and nicotinamide adenine dinucleotide phosphate (NADPH) oxidase (NOX) were all procured from the Jiancheng Bioengineering Institute in Nanjing, China. The concentrations of T‐SOD, GSH‐PX, MDA, mitochondrial complex I–V, ATP, and NOX in the heart and H9C2 cell homogenates were quantified using a visible spectrophotometer from Puxi Tongyong Instrument Co. Ltd., Beijing, China.

### 2.8 Western Blot

Western blot analyses were conducted in accordance with the previously reported reference [16]. The left ventricles from the border zone of the infarcted area in mice with MI and those from the corresponding zone in sham‐operated mice were excised and rapidly frozen in liquid nitrogen. Tissue and cell lysates were prepared using a lysis buffer composed of 20 mM Tris, 150 mM NaCl, 10% glycerol, 20 mM glycerophosphate, 1% NP40, 5 mM ethylenediaminetetraacetic acid (EDTA), 0.5 mM ethylenebis(oxyethylenenitrilo)tetraacetic acid (EGTA), 1 mM Na3VO4, 0.5 mM phenylmethanesulfonyl fluoride (PMSF), 1 mM benzamidine, 1 mM dl‐dithiothreitol (DTT), 50 mM NaF, 4 mM leupeptin, adjusted to pH 8.0. Equal quantities of total proteins, each at a concentration of 50 μg, were separated using 10% sodium dodecyl sulfate‐polyacrylamide gel electrophoresis (SDS‐PAGE) and subsequently transferred onto polyvinylidene fluoride (PVDF) membranes (Millipore, Billerica, MA, USA). The membranes were blocked using 5% non‐fat milk in Tris Buffered Saline with Tween (TBST) (comprising 50 mM Tris, 150 mM NaCl, and 0.5 mM Tween‐20, with a pH of 7.5), followed by an overnight incubation with primary antibodies. NOX2, phospho‐signal transducer and activator of transcription 3 (STAT3)‐Y705, and STAT3 were generated by ABclonal Technology. Glyceraldehyde‐3‐phosphate dehydrogenase (GAPDH) and corresponding secondary antibodies were sourced from Proteintech. Image J software (developed by NIH) was utilized for conducting densitometric analysis.

### 2.9 Reactive Oxygen Species (ROS) and Mitochondrial Membrane Potential (MMP) Determination [18]

Resuspend the H9C2 cell pellet in 1 mL of phosphate‐buffered saline (PBS), then add 10 μM of 2′,7′‐Dichlorodihydrofluorescein diacetate (DCFH‐DA) solution directly. Incubate the cells in a CO_2_ incubator at 37°C for 30–60 min. Following incubation, cells should be washed 2–3 times to eliminate any potential extracellular fluorescent substances and minimise background interference. Subsequently, resuspend the cells in a buffer solution, adjusting the cell density to 5 × 10^5^ cells/mL. Transfer 1 mL of this cell suspension into a tube containing a total of 10^4^ cells, and proceed to analyse using flow cytometry, with an excitation wavelength of 488 nm and an emission wavelength of 525 nm.

Wash the H9C2 cells with PBS, centrifuge, and collect the pellet. Resuspend the cells in 1 mL of PBS and incubate with a JC‐1 probe for 30 min. Subsequently, wash the cells with PBS again, centrifuge, and collect the pellet. Finally, resuspend the cells in 1 mL of PBS for further testing. Flow cytometry was used to detect cell apoptosis (Ex = 488 nm; Em = 530 nm), and green fluorescence was detected through the FITC channel, usually FL1; Red fluorescence was detected through the PI channel, usually FL2. For normal cells (FL‐1 bright, FL‐2 bright; R1), and apoptotic cells (FL‐1 bright, FL‐2 dark; R2), the position of the gate varies depending on cell type, experimental conditions, etc. The experiment necessitates the inclusion of untreated normal cells as a negative control group, as well as a positive control group. The gate position is established in accordance with the dual‐parameter scatter plot derived from these control groups. The MMP is determined by the ratio of fluorescence intensities between FL2 and FL1.

The ROS and MMP assay kits were both acquired from the Jiancheng Bioengineering Institute, located in Nanjing, China.

### 2.10 Quantitative Real‐Time Polymerase Chain Reaction (PCR) and Dual‐Luciferase Reporter Assay [19]

H9C2 cell lines were intervened with DMSP+PAE (100 μM) for 24 h (*n* = 6), and the control group was only intervened with DMSO (*n* = 6). After harvesting these samples, total RNA was extracted from H9C2 cell lines with TRIzol reagent (Invitrogen) according to the manufacturer's instructions. The RNA concentration in each sample was quantified using a NanoDrop spectrophotometer (Thermo). A total of 2 μg of RNA was reverse transcribed into cDNA using a Reverse Transcription Kit (Takara). Real‐time PCR was conducted with SYBR Green PCR Master Mix (Takara). The extraction process for total RNA from mouse heart tissue was analogous to the aforementioned method. The actin gene served as the internal control. The primers utilised are listed below: NOX2: forward primer 5'‐GCTTGTGGCTGTGATAAGCA‐3', reverse primer 5'‐ACGGCACAGCCAGTAGAAGT‐3'; GAPDH: forward primer 5'‐CTACCCACGGCAAATTCCAC‐3', reverse primer 5'‐CGGGCGTTGATGACAAGTTTCCCG‐3'; actin: forward primer 5'‐TACAACCTCCTTGCAGCTCC‐3', reverse primer 5'‐GGATCTTCATGAGGTAGTCAGTC‐3'.

The H9C2 cell lines were seeded into a 24‐well culture plate the day prior to transfection, with a cell density ranging from 70% to 80% at the time of transfection. A volume of 2 μL of lipo2000 was diluted into 50 μL of serum‐free Dulbecco's Modified Eagle's Medium (DMEM), while the plasmids were also diluted in 50 μL of serum‐free DMEM and allowed to incubate at room temperature for 20 min. Subsequently, serum‐free DMEM was added to reach a total volume of 100 μL. The culture medium was aspirated from the dish, and subsequently, 200 μL of the transfection complex, previously prepared, was introduced and incubated at 37°C for a duration of 5 h. Following this, the medium was discarded and replaced with 0.5 mL of complete medium, which was then incubated at the same temperature for a period of 48 h. The transfection process was repeated a total of five times. The transfection plasmid combinations were as follows: (1) DMSO with pGL3‐Basic; (2) PAE with pGL3‐Basic; (3) DMSO with the pGL3‐NOX2 promoter; (4) PAE with pGL3‐NOX2 promoter; (5) DMSO with the pGL3‐NOX2 promoter [delta1 (TTCCAGGATA) deletion]; (6) PAE with the pGL3‐NOX2 promoter [delta1 deletion]; (7) DMSO with the pGL3‐NOX2 promoter [delta2 (CACCAGGAAA) deletion]; (8) PAE with the pGL3‐NOX2 promoter [delta2 deletion]; (9) DMSO with the pGL3‐NOX2 promoter [delta3 (CTGCAGGAAG) deletion]; (10) PAE with the pGL3‐NOX2 promoter [delta3 deletion]. Lysate cells were harvested from a centrifugation at 10,000 g, and after spinning for 5 min, the supernatant was collected as the test solution. Subsequent operations were conducted in accordance with the protocol provided by the dual luciferase assay kit. When using marine luciferase as an internal control, the relative light units (RLU) measured for firefly luciferase were divided by the RLU values obtained from marine luciferase. Based on the calculated ratio, the activation level of the target reporter gene was compared across various samples.

### 2.11 Statistical Analysis

Data are expressed as means ± SEM values. Statistical analyses were performed by using SPSS version 20 (SPSS Inc., Chicago, IL). Tukey's post hoc test was employed to compare two groups subsequent to a one‐way ANOVA with a *p*‐value of less than 0.05. A *p*‐value less than 0.05 was deemed indicative of statistical significance.

## 3 Results

### 3.1 PAE Improved Heart Dysfunction and Remodelling in MI Mice

**FIGURE 1:**
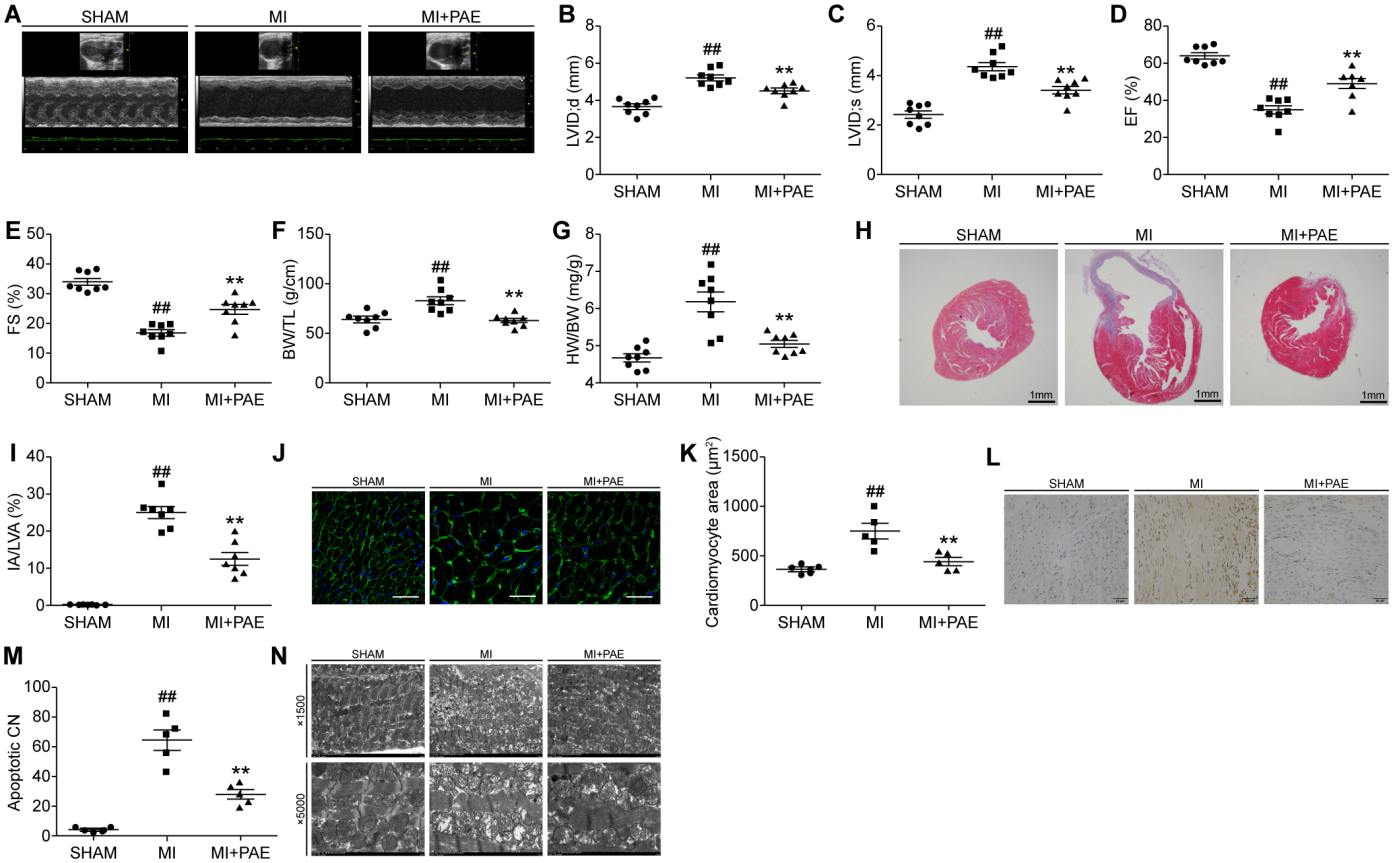
PAE improved heart dysfunction and remodelling in MI mice. (A–E) Echocardiography measurement (*n* = 8). (F) HW to TL ratio (*n* = 8). (G) HW to BW ratio (*n* = 8). (H) Masson staining. (I) Quantitative analysis (*n* = 7); scale bar: 1 mm. (J) WGA staining (400×). Scale bar: 50 μm. (K) Quantitative analysis (*n* = 5). (L) TUNEL immunohistochemistry staining (400×), scale bar: 50 μm. (M) Quantitative analysis (*n* = 5). (N) Electron microscopy detection. BW, body weight; CN, cardiomyocyte number; EF, ejection fraction; FS, fractional shortening; HW, heart weight; IA, infarcted area; LV, left ventricular; LVA, left ventricular area; LVID; d, left ventricular internal diameter at end‐diastole; LVID; s, left ventricular internal diameter at end‐systole; MI + PAE, myocardial infarction + paeonol group; MI, myocardial infarction group; SHAM, Sham‐Operation Group; TL, tibial length; TUNEL, terminal‐deoxynucleotidyl transferase‐mediated nick end labeling; WGA, wheat germ agglutinin. Data are given as means ± SEM. ^##^ *p* < 0.01 vs. SHAM group; **p < 0.01 vs. MI group.

Four weeks following MI, the heart function was significantly impaired, as evidenced by measurements of LVEF and LVFS, which were markedly lower than those of the sham group (*p* < 0.05). Furthermore, LVIDs and LVIDd in the MI group were significantly higher compared to the SHAM group (*p* < 0.05). Administration of PAE significantly improved the aforementioned 4 indicators 4 weeks following the MI (*p* < 0.05) (Figure 1A–E).

Among the three groups, the HW to TL ratio and HW to BW ratio were highest in the MI group, suggesting that MI induced heart hypertrophy (*p* < 0.05). Treatment with PAE for 4 weeks significantly inhibited heart hypertrophy after MI, as indicated by the decrease in the HW to TL ratio and HW to BW ratio (*p* < 0.05) (Figure 1F,G).

After intervening with PAE in normal mice for 4 weeks, there were no significant differences in heart function, the HW to TL ratio, and the HW to BW ratio compared to the control mice (*p* > 0.05) (Figure S1A–G).

Four weeks post MI, the infarcted area, as well as the size and apoptosis of cardiomyocytes in the border zone, were notably larger in the MI group compared to the sham group (*p* < 0.05). Upon treating MI mice with PAE, the aforementioned three indicators were significantly improved (*p* < 0.05) (Figure 1H–M).

Within the sham group, the mitochondrial membrane structures remained intact, exhibiting circular or elliptical shapes, and were densely and orderly arranged. The organisation of the mitochondrial cristae and matrix was uniform and distinct. In contrast, the mitochondria of the MI group exhibited a scattered arrangement, obvious swelling, a loose matrix, and evident partial fracture cristae. PAE significantly improved the abnormal structure of the mitochondria shown in the MI group (Figure 1N).

The findings demonstrated that PAE could mitigate heart dysfunction and cardiac remodelling in mice with MI.

### 3.2 PAE Reduced NOX2 Activity and Oxidative Stress, Maintained Mitochondrial Function, and Improved Cardiomyocyte Apoptosis and Collagen Deposition in the Hearts of MI Mice

**FIGURE 2:**
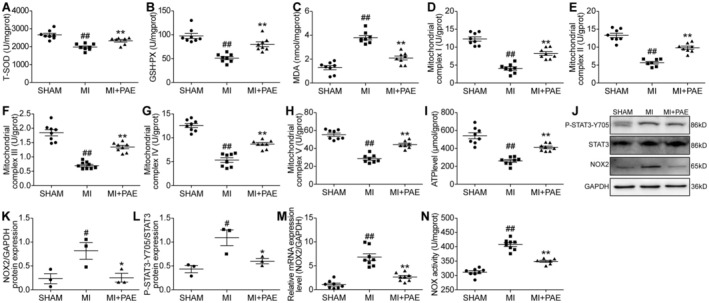
PAE reduced NOX2 activity and oxidative stress, maintained mitochondrial function, and improved cardiomyocyte apoptosis and collagen deposition in the hearts of MI mice. (A) T‐SOD activity (*n* = 8). (B) GSH‐Px activity (*n* = 8). (C) MDA level (*n* = 8). (D‐H) Activities of mitochondrial complex I, II, III, IV, and V (*n* = 8). (I) ATP level (*n* = 8). (J) Western blot analysis. (K, L) Quantitative analysis (*n* = 3). (M) NOX2 mRNA expression (*n* = 5). (N) NOX activity (*n* = 8). ATP, adenosine triphosphate; GAPDH, glyceraldehyde‐3‐phosphate dehydrogenase; GSH‐P_X_, glutathione peroxidase; MDA, malonaldehyde; NOX, nicotinamide adenine dinucleotide phosphate oxidase; STAT3, signal transducer and activator of transcription 3; T‐SOD, total‐superoxide dismutase. Data are given as means ± SEM. ^##^ *p* < 0.01, ^#^ *p* < 0.05 vs. SHAM group; **p < 0.01, *p < 0.05 vs. MI group.

Elevated levels of MDA and NOX activity, coupled with reduced T‐SOD and GSH‐Px activities in the hearts, confirmed the induction of cardiac oxidative stress in mice with MI, with statistical significance (*p* < 0.05). The administration of PAE to MI mice led to a reduction in MDA levels and NOX activity, while simultaneously enhancing T‐SOD and GSH‐Px activities. These findings indicate that PAE exhibits anti‐oxidative properties within the hearts of MI mice, as evidenced by statistical significance (*p* < 0.05) (Figure 2A–C,N).

Four weeks MI, the activities of mitochondrial complexes I, II, III, IV, and V, as well as the ATP content, were found to be significantly reduced (*p* < 0.05). However, PAE led to a significant enhancement in the activities of mitochondrial complexes and ATP levels (*p* < 0.05) (Figure 2D–I).

The expression levels of NOX2 and phospho‐STAT3‐Y705 proteins were markedly elevated in the myocardium of mice subjected to MI when compared to those in the sham‐operated group (*p* < 0.05). Administration of PAE effectively attenuated the protein expression levels of NOX2 and phospho‐STAT3‐Y705 in the hearts of MI mice (*p* < 0.05) (Figure 2J–L). Furthermore, the expression of NOX2 mRNA was found to be significantly elevated in the myocardium following MI, a phenomenon that was notably suppressed by treatment with PAE (*p* < 0.05) (Figure 2M).

The findings indicated that PAE was capable of diminishing NOX2 activity and oxidative stress, preserving mitochondrial function, and improving cardiomyocyte apoptosis and collagen deposition in the hearts of mice with MI.

### 3.3 PAE Improved Cell Remodelling and Its Mechanism in H9C2 Cells After LN Intervention

**FIGURE 3:**
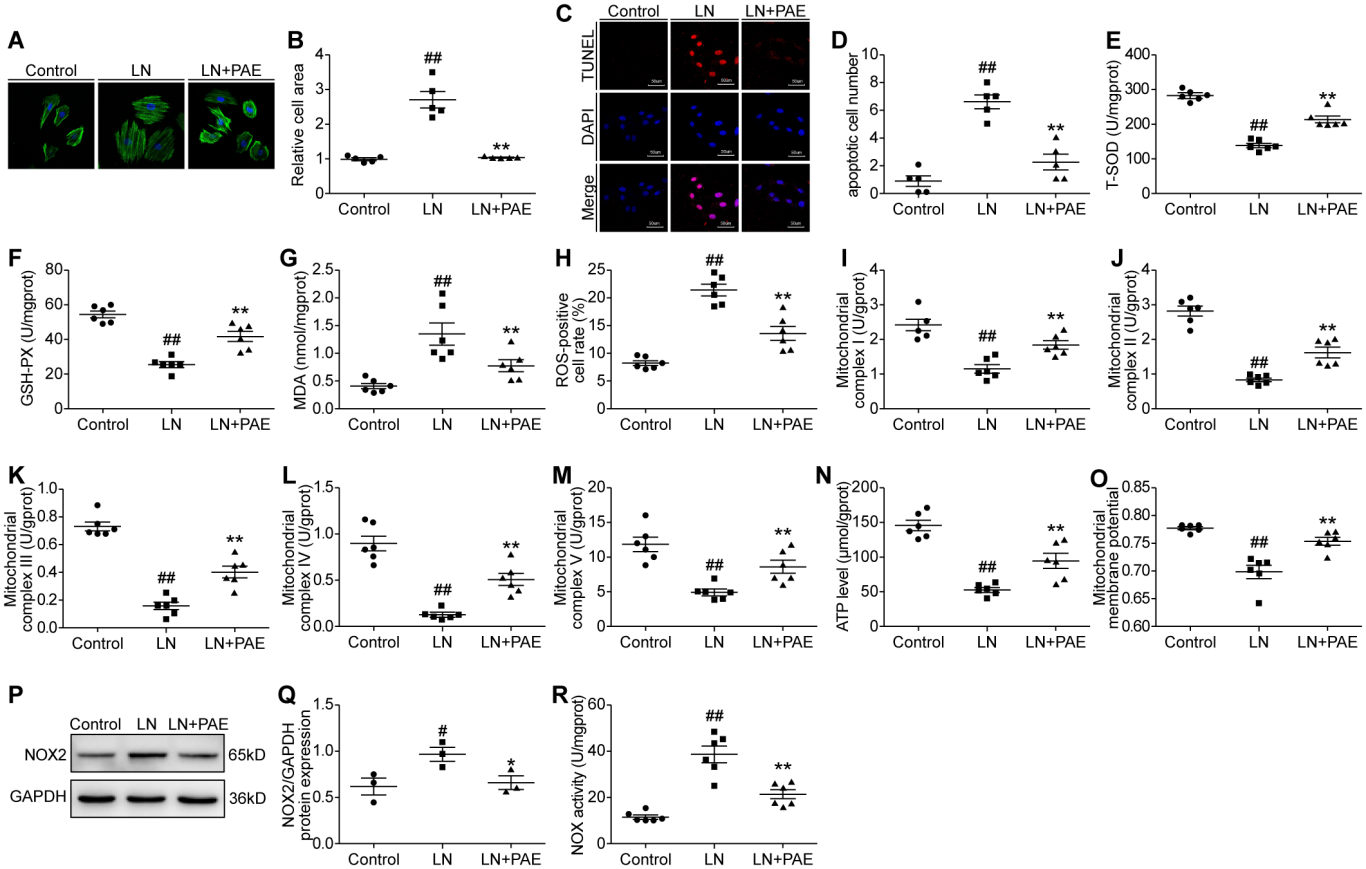
PAE improved cell remodelling and its mechanism after LN intervention. (A) Phalloidin staining (400×). (B) Quantitative analysis (*n* = 5). (C) TUNEL staining (400×), scale bar: 50 μm. (D) Quantitative analysis (*n* = 5). (E) T‐SOD activity (*n* = 6). (F) GSH‐Px activity (*n* = 6). (G) MDA level (*n* = 6). (H) ROS level (*n* = 6). (I–M) Activities of mitochondrial complex I, II, III, IV, and V (*n* = 6). (N) ATP level (*n* = 6). (O) Mitochondrial membrane potential level (*n* = 6). (P) Western blot analysis. (Q) Quantitative analysis (*n* = 3). (R) NOX activity (*n* = 6). Control Group; LN, levarterenol; control, LN + PAE, Levarterenol + paeonol group; MMP, mitochondrial membrane potential; ROS, Reactive oxygen species. Data are given as means ± SEM. ^##^ *p* < 0.01, ^#^ *p* < 0.05 vs. control group, **p < 0.01, *p < 0.05 vs. LN group.

Hyperactivation of the sympathetic nervous system plays a crucial role in myocardial remodelling following MI. Consequently, we employed LN to provoke cardiomyocyte remodelling in a controlled in vitro setting, thereby investigating the effectiveness and specific mechanisms of PAE. Following a 48‐h intervention with LN on H9C2 cells, there was a significant increase in both cell size and the number of apoptotic cells in the LN group compared to the control group (*p* < 0.05), indicating that the in vitro model of cardiomyocyte remodelling had been effectively established. Over this time frame, we subjected the cells to PAE for a duration of 24 h, which notably ameliorated cellular hypertrophy and apoptosis, as evidenced by a statistically significant improvement (*p* < 0.05) (Figure 3A–D).

Furthermore, H9C2 cells were intervened with PAE for 24 h, and we found that cell size and apoptosis were not significantly altered compared to the control cells (*p* > 0.05) (Figure S2A–D).

PAE also markedly mitigated the oxidative stress caused by LN, as evidenced by a significant elevation in the activities of T‐SOD and GSH‐PX, and a reduction in the levels of MDA and ROS (*p* < 0.05) (Figure 3E–H). Compared to the control group, the activities of mitochondrial complexes (I, II, III, IV, and V), the ATP level, and MMP were significantly reduced in the LN group (*p* < 0.05), while the levels of these indices were notably increased in the LN + PAE group (*p* < 0.05) (Figure 3I–O).

Following 48 h of intervention with LN, there was a significant increase in NOX2 and NOX activity compared to the control group (*p* < 0.05) (Figure 3P–R). Conversely, PAE administration led to a reduction in NOX2 and NOX activity within the LN + PAE group (*p* < 0.05) (Figure 3P–R).

The findings demonstrated that PAE was capable of reducing NOX2 activity and oxidative stress, preserving mitochondrial function, and improving cell apoptosis and size in H9C2 cells intervened with LN.

### 3.4 The Mechanism of PAE Regulating NOX2 mRNA Expression in H9C2 Cells

**FIGURE 4:**
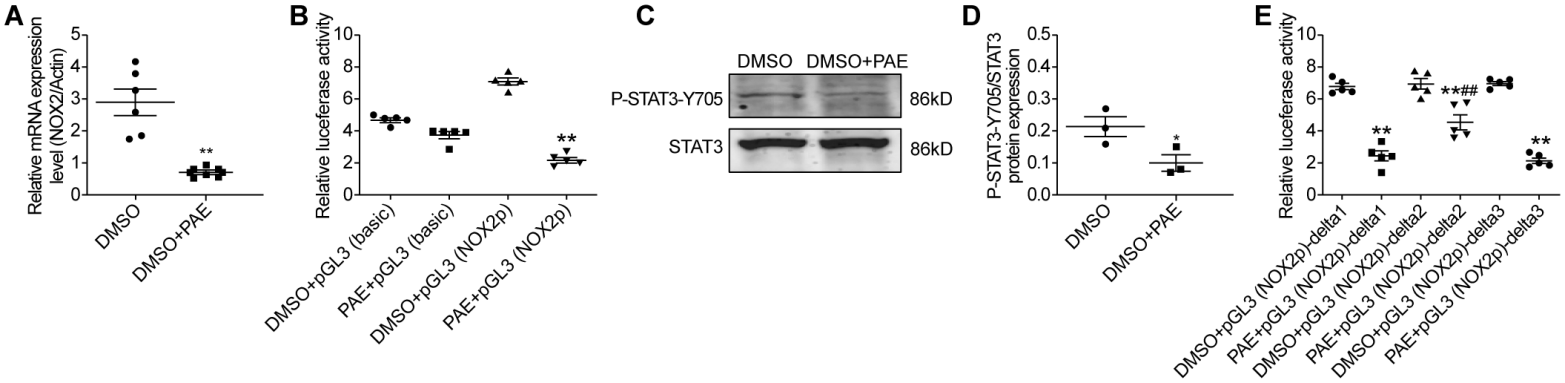
The mechanism of PAE regulating NOX2 mRNA expression in H9C2 cells. (A) Relative NOX2 mRNA expression (*n* = 6). (B) Dual luciferase assay (*n* = 5). (C) Western blot analysis. (D) Quantitative analysis (*n* = 3). (E) Dual luciferase assay (*n* = 5). DMSO: Dimethyl sulfoxide; NOX2p: NOX2 promoter. Data are given as means ± SEM. **p < 0.01 or *p < 0.05 vs. DMSO group or vs. DMSO + pGL3 (NOX2p) group or DMSO + pGL3 (NOX2p)‐delta1 group or DMSO + pGL3 (NOX2p)‐delta2 group or DMSO + pGL3 (NOX2p)‐delta3 group; ^##^ *p* < 0.01 vs. PAE + pGL3 (NOX2p)‐delta1 group or PAE + pGL3 (NOX2p)‐delta3 group.

Upon employing PAE to treat H9C2 cells for a duration of 24 h, we observed a significant down‐regulation of NOX2 mRNA expression in comparison to the control group (*p* < 0.05) (Figure 4A). To investigate its potential mechanism, we cloned the 5′ promoter region of the NOX2 gene in front of the Firefly luciferase gene to study the promoter activity in H9C2 cells. Upon treating H9C2 cells, which were previously transfected with the pGL3‐NOX2 promoter, with PAE, a marked reduction in dual‐luciferase activity was detected relative to the control group (*p* < 0.05) (Figure 4B).

We also found that phosphorylated STAT3 (Tyr 705) was significantly down‐regulated compared to the control group (*p* < 0.05) (Figure 4C,D). Subsequently, we utilised the Jaspar Database to analyse the potential binding sites within the 5′ promoter region of the NOX2 gene for STAT3. After deleting the delta1, delta2, or delta3 sequences, dual‐luciferase activity was significantly reduced compared to the control groups (*p* < 0.05). However, when compared to the delta1 or delta3 deletion groups, the dual‐luciferase activity in the delta2 deletion group was significantly increased (*p* < 0.05) (Figure 4E).

The findings suggested that PAE might exert a negative transcriptional regulation on NOX2 gene expression, partly through suppressing the phosphorylation of STAT3 (Tyr 705) in H9C2 cells.

## 4 Discussion

NOX is a transmembrane enzyme situated within intracellular organelles [20]. NOX catalyses the conversion of oxygen into superoxide, serving as the primary generator of reactive oxygen species (ROS). It is also the sole enzyme that directly produces ROS, which includes superoxide anions (O^2−^), hydrogen peroxide (H_2_O_2_), and hydroxyl radicals (OH‐), among others [20, 21]. Owing to their unpaired electrons, ROS exhibit a high degree of chemical reactivity [20, 21]. Under typical circumstances, ROS, which is a natural byproduct of oxygen metabolism, exists at a relatively low concentration within the body. It functions as an “oxidation–reduction messenger,” facilitating intracellular signal transduction and regulation. It plays a crucial role in sustaining the cell cycle, gene expression, and the homeostasis of the cellular environment [22]. However, when the body undergoes stimulation—be it from hypoxia, ischemia, radiation, or similar conditions—the concentration of ROS rises steeply, outstripping the body's natural capacity for clearance and processing. An imbalance between oxidation and antioxidation within the body leads to oxidative stress. Ultimately, lipid peroxidation ensues, alongside alterations in protein structure and function, cell membrane degradation, DNA damage, and eventually cell death [22, 23]. NOX2 is the primary enzyme responsible for generating ROS in HF, predominantly located in the cell membrane. It comprises membrane‐bound subunits gp91phox and p22phox [24]. Upregulation of NOX2 expression has been observed in the left ventricular myocardial tissue of HF patients [25]. The activity of NOX2 escalates following MI, and the genetic deletion of NOX2 exerts a protective effect against post‐MI cardiac remodelling [26]. In summary, these findings indicate that NOX2 could serve as a significant novel therapeutic target to safeguard against myocardial remodelling following MI.

In the current investigation, following MI for a duration of 4 weeks, we observed a marked increase in the expression of NOX2 mRNA and protein, which was significantly elevated compared to the sham‐operated group. Additionally, NOX activity was notably enhanced in the MI group. The findings suggested that the elevation in NOX activity was predominantly attributed to the overexpression of NOX2 mRNA and protein, which played a crucial role in the progression of cardiac remodelling following MI.

T‐SOD and GSH‐Px are pivotal components of the endogenous antioxidant defence system, playing a critical role in mitigating oxidative damage, eliminating free radicals, and preserving the redox balance within cardiomyocytes [27]. T‐SOD catalyses the dismutation of O_2_^−^ to H_2_O_2_ [27, 28]. GSH‐Px helps to metabolise H_2_O_2_ to non‐toxic products and prevents lipid peroxidation [27, 29]. Lipid peroxidation is marked by the presence of MDA, a compound that disrupts the permeability and fluidity of cell membranes, thereby impairing the structure and function of cardiomyocytes [30]. In this investigation, we observed a significant reduction in the activities of antioxidants, specifically T‐SOD and GSH‐Px, alongside a marked elevation in MDA levels. These findings suggest that oxidative stress ensues from a disruption in the equilibrium between antioxidant defences and oxidative agents. Oxidative stress may induce cardiac hypertrophy following MI by triggering signalling pathways that facilitate cardiac hypertrophy, thereby enhancing cardiomyocyte enlargement and extracellular matrix accumulation [31]. In our study, following a 4‐week period of MI in mice, we observed a significant increase in cardiomyocyte size, infarct size, and collagen deposition. These findings suggest that oxidative stress may play a substantial role in the progression of cardiac hypertrophy subsequent to MI. Additionally, oxidative stress is implicated in the induction of cardiomyocyte apoptosis during MI, as indicated by reference [32]. Our research findings were consistent with previously reported data. In summary, these findings indicated that the overactivation of NOX2 primarily contributed to oxidative stress, leading to cardiac hypertrophy and cardiomyocyte apoptosis in mice subjected to MI for a duration of 4 weeks.

Cumulative evidence indicates that PAE possesses the ability to inhibit oxidative stress and exerts a protective effect across a range of diseases, including atherosclerosis and diabetic cardiomyopathy [33, 34]. Nevertheless, the precise mechanism remains obscure. Our research revealed that PAE suppressed oxidative stress by transcriptionally inhibiting the expression of NOX2 mRNA in H9C2 cells. This new discovery has not been reported previously. Related references have reported that PAE inhibits the phosphorylation of STAT3, which is important for hepatic stellate cell activation and for the proliferation and metastasis of non‐small cell carcinoma [35, 36]. It has been reported that the phosphorylation of the tyrosine residue at position 705 on STAT3 enables it to form homodimers or heterodimers, and subsequently translocate to the nucleus. There, it binds to specific DNA sequences and activates the transcription of target genes [37]. In our study, we utilised the Jaspar Database to predict that STAT3 may bind to the promoter of the NOX2 gene and the potential binding site. Furthermore, the luciferase reporter gene experiment demonstrated that STAT3 can bind to the specific site of the NOX2 gene promoter, and PAE partially inhibited the expression of NOX2 mRNA by suppressing STAT3 (Tyr 705) phosphorylation. Due to the inhibition of NOX2‐induced oxidative stress, PAE exerted a protective effect against myocardial remodelling after MI in mice.

The excessive activation of the sympathetic nervous system plays a critical role in myocardial remodelling after MI [38]. Chronic stress results in prolonged exposure of the heart to norepinephrine, a neurotransmitter that is activated by α‐ and β‐adrenergic receptors. This exposure induces alterations in cardiac function and structure, including hypertrophy and apoptosis [39]. Cumulative data indicate that during sympathetic overexcitation, myocardial tissue initiates a cascade of signalling pathways, leading to an excessive production of ROS within cardiomyocytes or a disruption of the antioxidant defence mechanisms. This results in an imbalance between oxidative and antioxidative processes. Consequently, such disturbances can cause structural and functional abnormalities in cardiomyocytes, potentially leading to apoptosis of these cells [40, 41]. Hence, the in vitro model of cardiomyocytes stimulated with norepinephrine serves as a pertinent and suitable platform for assessing the cardioprotective efficacy of PAE in mitigating damage induced by hyperactivation of the sympathetic nervous system. In this investigation, we employed PAE to treat H9C2 cells that had been stimulated with LN. We subsequently confirmed that PAE can diminish cell size and inhibit apoptosis by specifically suppressing the overactivation of NOX2, thereby curbing oxidative stress in a laboratory setting. To date, the assessment of ROS levels has predominantly been conducted in vitro [42]. In this experiment, we employed a fluorescent probe to tag ROS‐positive cells, subsequently quantifying them via flow cytometry. The findings indicated that PAE effectively suppressed the rise in ROS levels triggered by LN in H9C2 cells, thereby implying that PAE has the potential to mitigate oxidative stress through a significant reduction in ROS overproduction under sympathetic overactivation.

During the catalytic process of ATP generation, the mitochondrial electron respiratory chain produces a small quantity of reactive oxygen species (ROS), with complexes I and III of the mitochondrial respiratory chain being the primary sources [43]. ROS has the ability to directly target mitochondrial respiratory chain complexes I and III, and when their function is compromised, it can lead to increased ROS production within the mitochondria. This, in turn, can further aggravate mitochondrial damage and oxidative stress [44]. In this study, the diminished activities of mitochondrial complexes I and III were primarily attributed to excessive production of ROS, a consequence of the overactivation of NOX2 in mice with MI or H9C2 cells exposed to LN stimulation. This ultimately exacerbated oxidative stress and caused damage to mitochondrial structure. Following the suppression of NOX2 activity through PAE treatment in these models, reduced ROS production led to the restoration of mitochondrial complexes I and III, improving mitochondrial‐induced oxidative stress and mitigating structural damage. Furthermore, the mitochondrial respiratory chain complexes I through V function in concert to convert phosphorylated ADP into ATP [43, 45]. Our research revealed that the activities of mitochondrial complex II, IV, and V were also significantly diminished as a result of ROS assault following MI for a duration of 4 weeks, or when H9C2 cells were intervened with LN. As a result, these groups exhibited lower ATP production compared to the normal groups. The suppression of ROS overproduction by PAE led to the revitalisation of mitochondrial complexes I–V, facilitating the smooth operation of mitochondrial energy metabolism in our study.

MMP serves as a reliable indicator that reflects the permeability of both the inner and outer mitochondrial membranes. It plays a crucial role in ATP production and in sustaining cellular homeostasis [46]. ROS compromises the integrity of the mitochondrial membrane, leading to a reduction or obliteration of ion gradients across it. This, in turn, causes a decrease or potentially a complete loss of MMP [46]. As a consequence, the mitochondrial respiratory chain's oxidative phosphorylation mechanism is disrupted, leading to a reduction or halt in ATP production. This disruption triggers the release of Ca^2+^ from the mitochondrial matrix, which ultimately induces cell apoptosis [46, 47]. As a result, LN may provoke an overabundance of ROS by activating NOX2 excessively, thereby inhibiting MMP and causing apoptosis in H9C2 cells as observed in our investigation. Following the inhibition of NOX2 by PAE treatment, H9C2 cells exhibited reduced ROS production, restored MMP, and decreased cell apoptosis subsequent to LN intervention.

Looi and his colleagues have reported that NOX2^−/−^ mice exhibited reduced left ventricular dilatation, cardiomyocyte hypertrophy, apoptosis, and interstitial fibrosis, and had better preserved systolic function following MI compared to their wild‐type littermates [26]. The findings suggest that the protective effect of PAE on myocardial remodelling following MI primarily stems from the inhibition of NOX2 activity. Furthermore, we treated normal mice with PAE for 4 weeks and H9C2 cells for 24 h, with no observed toxicity in vivo or in vitro, indicating that PAE is relatively safe.

## 5 Conclusions

**FIGURE 5:**
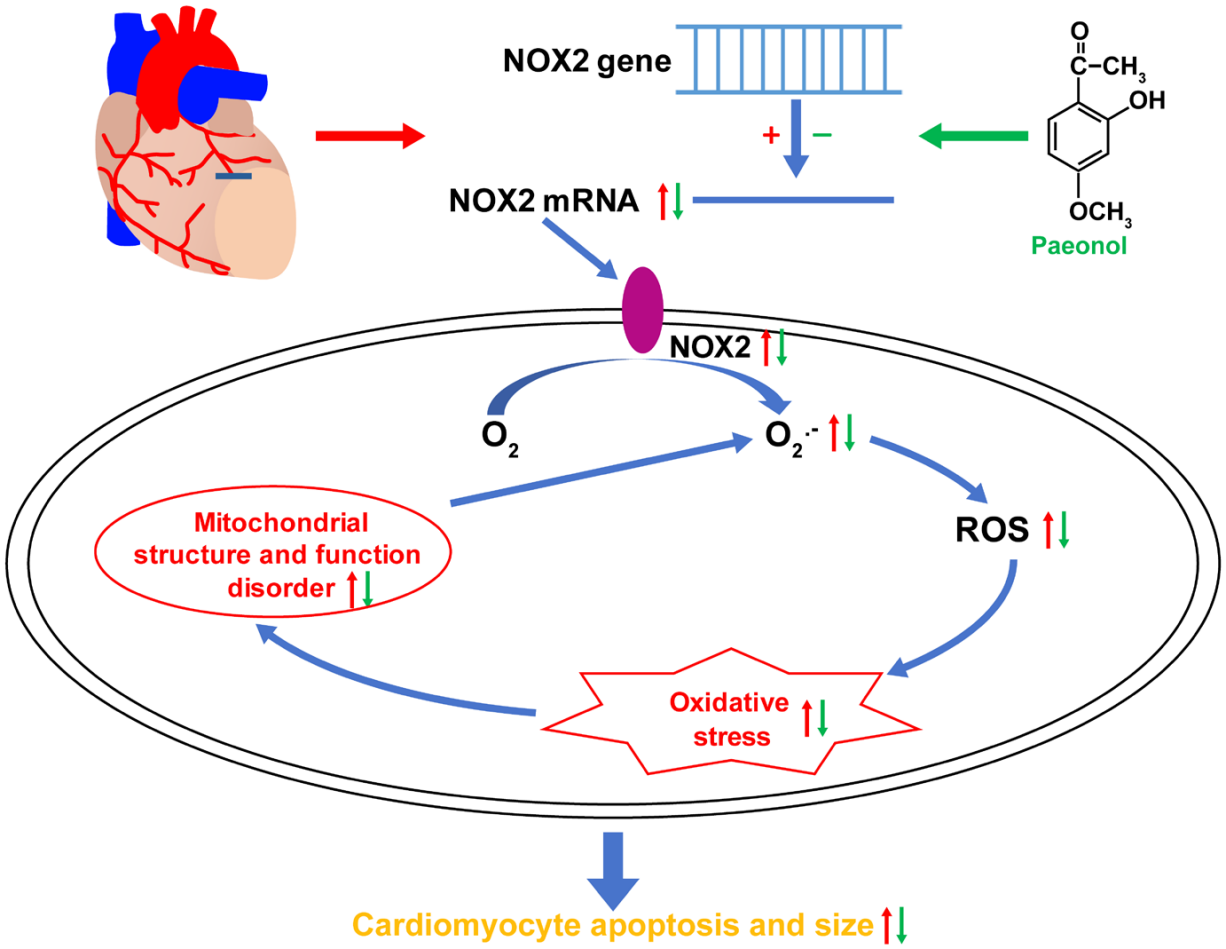
Schematic of the mechanism by which PAE improves myocardial remodelling in mice with MI. After complete ligation of LAD for a long time to induce cardiac remodelling in mice, mRNA and protein expressions of NOX2 in cardiomyocytes significantly increased, and the generation of O_2_^−^ and ROS caused by it significantly increased, leading to oxidative stress. On the one hand, it could increase cardiomyocyte apoptosis and hypertrophy, while on the other hand, it could damage the structure and function of mitochondria, leading to a decrease in ATP level and MMP, and promote the generation of more O_2_^−^, which could exacerbate oxidative stress. Eventually, it could further lead to cardiomyocyte apoptosis and hypertrophy and form a vicious cycle. The application of PAE in the treatment of these MI mice could inhibit the protein expression of NOX2 by transcriptional negative regulation of its mRNA formation, thereby inhibiting the above pathways, alleviating oxidative stress, and maintaining mitochondrial function, achieving the effect of reducing cardiomyocyte apoptosis and hypertrophy.

In conclusion, the overproduction of ROS from NOX2 can result in oxidative stress and mitochondrial dysfunction, playing a significant role in the development of cardiac remodelling following MI. Transcriptionally inhibiting NOX2 mRNA expression by PAE exerted a cardioprotective effect against myocardial remodelling post‐MI by improving oxidative stress and mitochondrial dysfunction. The findings demonstrated that PAE holds therapeutic promise for the treatment of patients suffering from ischaemic cardiomyopathy (Figure 5).

## Author Contributions

**Yun Liu:** formal analysis (equal), investigation (equal), methodology (equal). **Zhiming Wu:** formal analysis (equal), funding acquisition (equal), investigation (equal), methodology (equal). **Xiaoping jin:** formal analysis (equal), investigation (equal), methodology (equal). **Meili Ji:** investigation (equal), methodology (equal). **Tianyi Huang:** investigation (equal), methodology (equal). **Peina Meng:** investigation (equal), methodology (equal). **Tian Xu:** investigation (equal), methodology (equal). **Wei You:** conceptualization (equal), data curation (equal), project administration (equal), writing – review and editing (equal). **Yanfang Zhao:** conceptualization (equal), data curation (equal), project administration (equal), writing – review and editing (equal). **Fei Ye:** conceptualization (equal), data curation (equal), project administration (equal), writing – review and editing (equal). **Xiangqi Wu:** conceptualization (equal), data curation (equal), project administration (equal), supervision (equal), writing – original draft (equal), writing – review and editing (equal).

## Conflicts of Interest

The authors declare no conflicts of interest.

## Supporting information


**Figure S1:** Effect of PAE on heart function and heart weight in normal mice for 4 weeks. (A–E) Echocardiography measurement (*n* = 6). (F) HW to TL ratio (*n* = 6). (G) HW to BW ratio (*n* = 6).

**Figure S2:** Effect of PAE on cell size and apoptosis in H9C2 cells for 24 h. (A) Phalloidin staining (400×). (B) Quantitative analysis (*n* = 5). (C) TUNEL staining (400×). (D) Quantitative analysis (*n* = 5).

## Data Availability

The data that support the findings of this study are available on request from the corresponding author.
