# Supplementary figures and images for: Paeonol Improves Cardiac Remodelling in MI Mice by Suppressing NOX2 mRNA Expression to Mitigate Oxidative Stress and Mitochondrial Dysfunction
Source: J Cell Mol Med. 2025 May 8;29(9):e70563. doi: 10.1111/jcmm.70563 (PMC12061634; doi:10.1111/jcmm.70563)

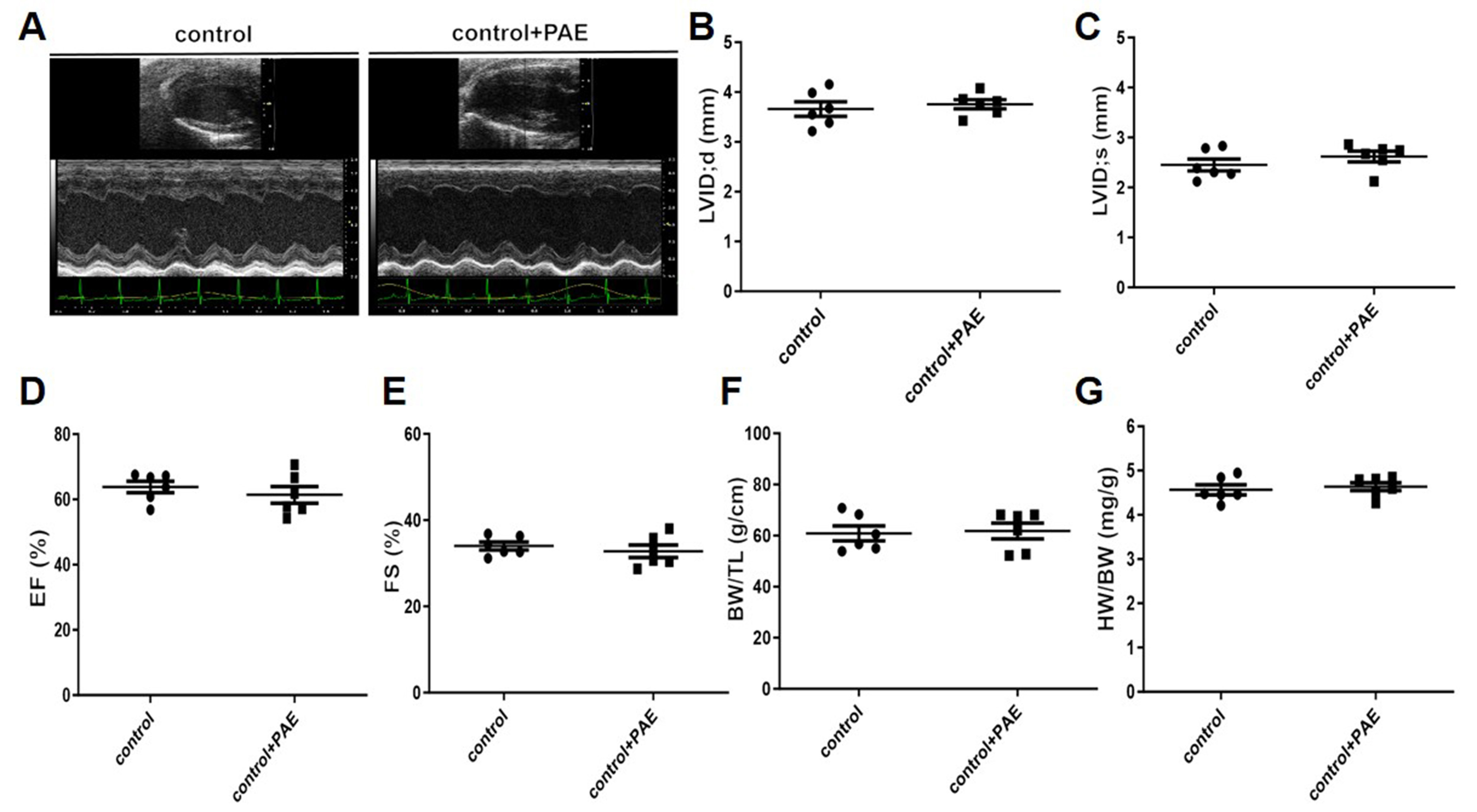

Supplement: Supplementary file 1 — Figure S1: Effect of PAE on heart function and heart weight in normal mice for 4 weeks. (A–E) Echocardiography measurement (n = 6). (F) HW to TL ratio (n = 6). (G) HW to BW ratio (n = 6). [file JCMM-29-e70563-s001.jpg]

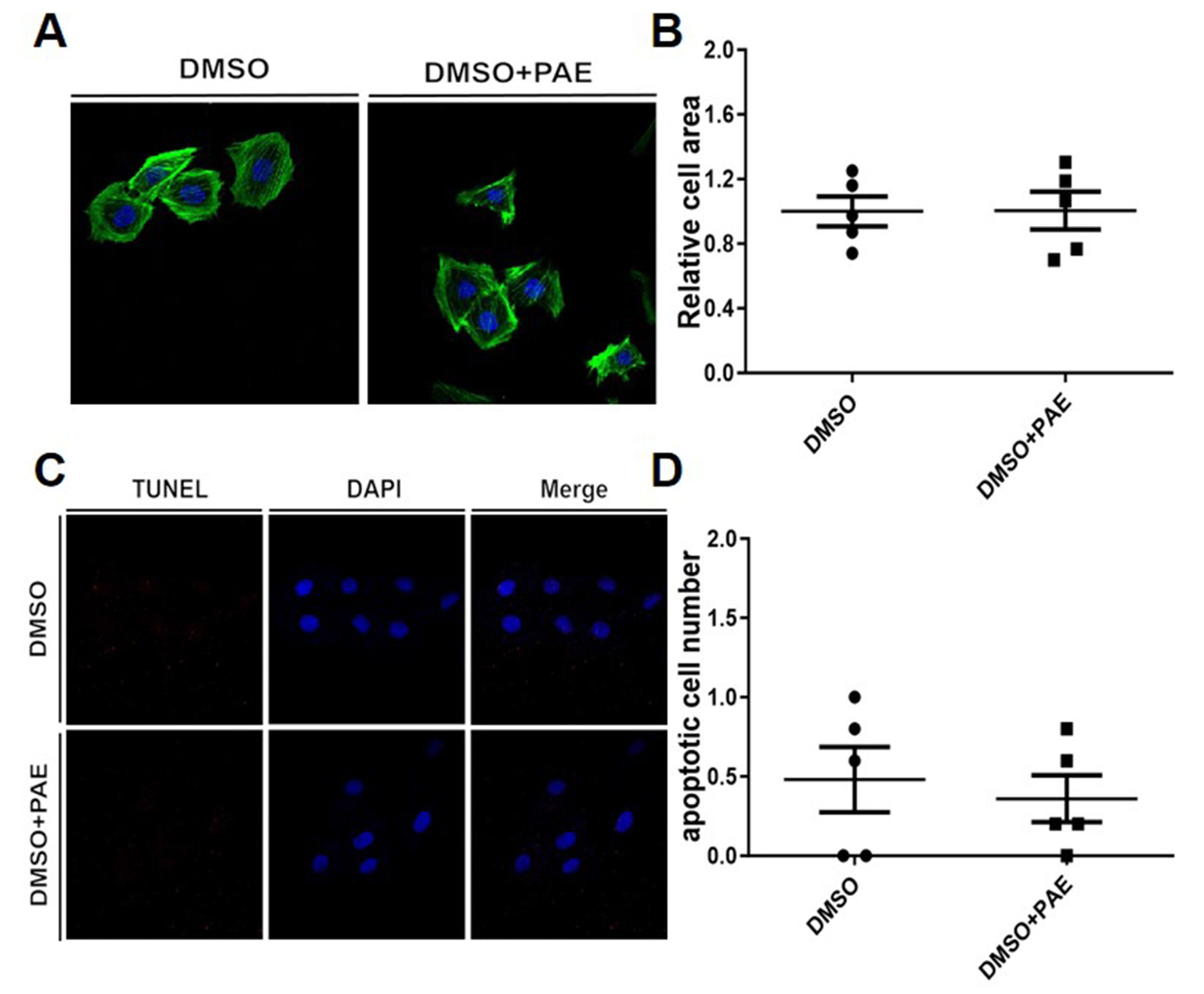

Supplement: Supplementary file 2 — Figure S2: Effect of PAE on cell size and apoptosis in H9C2 cells for 24 h. (A) Phalloidin staining (400×). (B) Quantitative analysis (n = 5). (C) TUNEL staining (400×). (D) Quantitative analysis (n = 5). [file JCMM-29-e70563-s002.jpg]
